# The Temporal Association between Body Characteristics and Speed Performance over Twenty-Five Years in Italian Adolescents

**DOI:** 10.3390/children9040521

**Published:** 2022-04-06

**Authors:** Matteo Vandoni, Vittoria Carnevale Pellino, Annalisa De Silvestri, Nicola Lovecchio, Antonio Rovida, Alessandro Gatti, Valentina Biagioli, Gianvincenzo Zuccotti, Valeria Calcaterra

**Affiliations:** 1 Laboratory of Adapted Motor Activity (LAMA), Department of Public Health, Experimental Medicine and Forensic Science, University of Pavia, 27100 Pavia, Italy; vittoria.carnevalepellino@unipv.it (V.C.P.); antonio.rovida@unipv.it (A.R.); alessandro.gatti08@universitadipavia.it (A.G.); valentina.biagioli01@universitadipavia.it (V.B.); 2 Department of Industrial Engineering, University of Rome Tor Vergata, 00133 Rome, Italy; 3 Biometry and Clinical Epidemiology, Scientific Direction, Fondazione IRCCS Policlinico San Matteo, 27100 Pavia, Italy; a.desilvestri@smatteo.pv.it; 4 Department of Human and Social Science, University of Bergamo, 24127 Bergamo, Italy; nicola.lovecchio@unibg.it; 5 Department of Pediatrics, “Vittore Buzzi” Children’s Hospital, 20157 Milano, Italy; gianvincenzo.zuccotti@unimi.it (G.Z.); valeria.calcaterra@unipv.it (V.C.); 6 Department of Biomedical and Clinical Science “L. Sacco”, University of Milano, 20157 Milano, Italy; 7 Pediatric and Adolescent Unit, Department of Internal Medicine and Therapeutics, University of Pavia, 27100 Pavia, Italy

**Keywords:** physical fitness decline, obesity, Italian adolescents, speed-agility trend

## Abstract

Background: Physical fitness (PF) is positively related to skeletal and metabolic health, and it had an inverse relation with obesity. Adolescents with obesity have the worst performance in PF and speed-agility (SA) that contributes to an augmented risk to develop pathologies. To the best of our knowledge, many studies analyzed the trends of obesity and SA separately, but there is a lack of data about SA ability trends in adolescents with obesity. We aimed to investigate SA trends in children with obesity in the last few decades to define the association between body weight and physical performance. Methods: We recruited 3.923 Caucasian children across the period 1985–2010 in the same school in Northern Italy, near Milan. Once a year, at the ages of 11–12- and 13-years-old, we collected anthropometric measures and SA performance. We pooled the data into 5-year-period study waves and then stratified our analysis into test-sex-age BMI-z-score specific groups. Results: We reported an undetermined trend across years. The 4×5 m run test significantly decreased in adolescents with overweight/obesity, while we did not report a decline in 30 m and 60 m run tests. Conclusions: Fitness tests highlighted differences in normal weight compared to overweight/obese children, suggesting that it is crucial to carefully monitor PF capacities through the years.

## 1. Introduction

The World Health Organization [1] reported an increase in the prevalence of children and adolescents (5–19 years old) with an overweight (OW) body mass index (BMI) from 1975 to 2016; and, in Italy, the National Health Institute declared that over 29% of school-age children are OW and more than 10% are affected by obesity [2]. Children and adolescents with obesity have an increased risk of developing cardiovascular and metabolic diseases, such as insulin resistance, type 2 diabetes, coronary disease, and hypertension [3]; and they tend to be adults with obesity, with an augmented rate of co-morbidities and mortality [4]. Physical activity (PA) and active lifestyle promotion fill a fundamental role in reducing the negative effects of weight gain and cardio-metabolic pathologies in children and adolescents [5,6]. 

On the other hand, Physical Fitness (PF) is the ability to perform an exercise with the correct development of physiological and psychological skills [7,8], and previous studies have shown that a poor level of PF should be identified as a “warning sign” in children’s health [9]. In fact, PF has an inverse relation with obesity and the risk of CVD, and it is also positively related to skeletal and metabolic health [7,10]. 

Unfortunately, adolescents with obesity have a lower PF compared to their normal-weight peers [11,12,13,14], with a direct repercussion on the engagement and the adherence to PA practice [15]. An assessment of PF is composed of multiple physical capacities such as cardiorespiratory endurance, muscular strength, and speed ability (SA). Cardiorespiratory endurance has been inversely related to CVD risk, adiposity, low self-esteem, cancer, and all-cause mortality in late life [16,17,18]. Several studies showed a decline of cardiorespiratory endurance in children, and a study by Johansson et al. [19] showed a decline of this component in children with obesity. Muscular strength is also inversely related to CVD risk and mortality, insulin resistance, bone health, and adiposity [20]. A study by Đurić et al. [21] showed a decrease in arm strength and leg power while the core muscle strength increased from 1983 to 2014. The SA ability is defined as a combination of the ability to move quickly and to change direction, maintaining control and balance [22]; and, in particular, we can portray it as the biomechanical performance of the strength of the lower limbs that are influenced by one’s own weight [23]. The SA is considered as a key marker of bone health in young people, and it is usually associated with enhanced performance in sports [24], highlighting the importance of testing this capacity through the years. To evaluate SA in children and adolescents, several studies showed the use of the 30-m sprint test and the 4×10 m shuttle run test as simple, accurate, and reliable methods [7,25,26,27].

In general, some evidence showed a PF decline through the years. Ogden et al. [28] found that the prevalence of children with obesity increased in the U.S. This trend was also confirmed by Fan et al. [29]. Eberhardt et al. [30] reported an overall decline in PF components, except for the SA ability. Additionally, Fühner et al. [31] showed a significant decrease of cardiorespiratory endurance from 1972 to 2015, reporting a small increase in the SA ability. To the best of our knowledge, many studies analyzed the trends of obesity and SA separately [28,29,30,31], but there is a lack of data about trends in SA ability in adolescents with obesity. In fact, cardiorespiratory fitness had a moderately negative association with body mass, waist circumference and body fatness (r = −0.52), while SA had a low negative association with fat mass and BMI (r = −0.24) [15,32,33]. The SA ability trends remain unclear in children and adolescents with obesity and the evaluation of a single PF component related to physical health [15,33,34,35], so, the aim of this study was to investigate the trends of SA in children with obesity in the last decades in order to define the role of body weight on physical performance maintenance. In particular, we take into account three different tests largely used to assess SA because physical demands could be crucial factors in evaluating the abilities of the young during growth. To our knowledge, this is the first study to examine the temporal trends of SA in children with obesity in an Italian population.

## 2. Materials and Methods

### 2.1. Sample and Study Design 

We conducted a cross-sectional study on a total of 3.923 Caucasian adolescents aged 11–13 years, who were recruited across the period 1985–2010, from a single middle-high school in northern Italy, near Milan. According to the WHO classification BMI z-score [36], adolescents were divided into two groups: normal-weight (−2 ≤ BMI-z score ≤ 1) and overweight/with obesity when a BMI-z score was > 1/2. The inclusion criteria were adolescents aged 11 to 13 years old of both genders, with no previously known neurological/orthopedic or cardiovascular diseases and no illness that could affect growth and actively attending school physical education (PE) classes. The exclusion criteria were reported orthopedic injuries in the last six months, any condition that did not permit participation in curricular PE classes, and having any medical condition that could affect exercise participation. Parents or legal guardians provided written informed consent after the explanation of the study procedures. Adolescents provided verbal absence, and they were informed that their participation was voluntary and that they could withdraw at any time without prejudice. No academic credit or benefit was given to adolescents for participation. The study was conducted in accordance with the Declaration of Helsinki as revised in 2018 [37]. The study protocol was approved by the institutional review board of Regione Lombardia (D.g.r. 9 June 2017—n. X/6697) along with the Italian National Olympic Committee (CONI).

### 2.2. Testing Procedures

Over the 25-year period, at the same time of day (8:00 am to 1:00 pm) and once a year (between mid-September and mid-October), participants were tested by the same teacher during two consecutive PE classes. Weight and height were measured without shoes and in light clothes using procedures described by the International Society for the Advancement of Kinanthropometry [38]. Weight was measured using a balance scale to the nearest 0.1 kg. Height was measured with a stadiometer to the nearest 1 cm, with participants standing upright and their heads in the Frankfort plane. Body Mass Index (BMI) was calculated using the following formula: body mass (kg) divided by squared height (m^2^). 

### 2.3. Physical Fitness Test 

The data collection consisted of a series of physical fitness tests selected within the widespread Eurofit battery [39] and from usual practice considering the school settings conditions [23,40,41]. All data were collected by one PE teacher in full compliance and collaboration with the curricular PE classes. To evaluate SA in adolescents, several studies showed the use of the 30-m sprint test and the 4×10 m shuttle run test as simple, accurate, and reliable methods [7,25,26,27]. These field tests are reliable and valid instruments to measure physical fitness in children and adolescents [15,41], and they are strictly defined, simple to administrate, and cheap to use [22]. 

### 2.4. 30-m Sprint Test 

During PE lessons in the school gym, the participant performed a 30 m sprint, running at a maximum speed, (ICC = 0.96) [42]. The start and endpoints were marked on the floor with a colored scotch to ensure the correct distance measurements. The same operator started the chronograph after the phrase “3, 2, 1, go”; and, they stopped the chronograph when the child crossed the end line with one foot. The time was recorded using a chronograph (Stopwatch W073, SEIKO, Tokyo, Japan), with a time resolution of 0.01 s. A higher time indicated a worse performance. Before starting the test, the evaluator performed an example to ensure that the adolescents understood the test procedure correctly. 

### 2.5. 60-m Sprint Test

During PE lessons in the school gymnasium, the participant performed a 60 m sprint test, running at a maximum speed. The start and the endpoints were marked on the floor with a colored scotch to ensure the correct measurement of the distance. The same operator started the chronograph after the phrase “3, 2, 1 go”; and, they stopped the chronograph when the child crossed the end line with one foot. The time was recorded using a chronograph (Stopwatch W073, SEIKO, Tokyo, Japan), with a time resolution of 0.01 s. A higher time indicated a worse performance. Before starting the test, the evaluator performed an example to ensure that the adolescents understood the test correctly. 

### 2.6. 4 m × 5 Run Test

This test consisted of running and turning as fast as possible between two parallel lines (5 m apart), where adolescents had to exchange sponges when crossing the lines (4 times of 5 m; ICC = 0.90–95% CI 0.75–0.97) [43]. This test was used to investigate speed-agility [22]. The result was measured with a stopwatch (Stopwatch W073, SEIKO, Tokyo, Japan) to the nearest 0.1 s by the same evaluator [43]. The best of two attempts was recorded in seconds. A higher time indicated a worse performance. Before starting the test, the evaluator performed an example to ensure that the adolescents understood the test correctly. A slip-proof floor, four cones, a stopwatch, and three sponges were used to perform the test.

### 2.7. Statistical Analysis

All data were manually recorded from the PE teacher, then inserted into a spreadsheet and checked for transcription errors, with corrections made where appropriate by three different expert operators. The dataset was also checked for outliers, with minimum and maximum time outliers not attained for the tests (4×5 m, 30-m, 60-m). Quantitative variables were provided as mean ± standard deviations (SD) or percentages as appropriate. To compare trends across different tests, sexes, ages and z-score BMI categories (normal weight adolescents and adolescents with overweight/obesity in accordance with WHO stratifications) and trends from the published literature, we first pooled the data into 5-year study waves [44,45] (e.g., <1990, 1990–1995, 1995–2000, 2000–2005 and 2005–2010) and then stratified our analysis into test-sex-age-BMI z-score specific groups (e.g., 11-year-old girls normal-weight tested on the 4×5 m, 11-year-old girls overweight tested on the 4×5 m, etc.). During the observation period, PF performances were analyzed by fitting multilevel (subjects and school classes) mixed effect linear regression models in which the random portion consisted in subjects (thus correcting for individual variation) and classes. The fixed portions were represented by calendar years (5-year periods), age, sex, and BMI classes. The models are random intercept allowing each subject to have a separate intercept; thus, it is possible to consider the dependency of the data (obtaining the correct standard errors) and to adjust for individual factors (measured and unmeasured confounders). In this repeated cross-sectional design, multilevel models take into account the clustered nature of the data within individuals and classes. Measurement occasions form the level 1 units and individuals are shifted up to level 2 and classes at level 3. The results are expressed as coefficients with their 95% confidence interval (95% CI), and they are presented with term-specific *p* values; the coefficient represents the mean variation of outcomes for unit change of quantitative predictors or between levels of categorical or ordinal predictors. Since a higher time indicated a worse performance, a negative coefficient indicated a better performance, and a *p* < 0.05 was considered statistically significant. Data analyses were performed with STATA v16.1 (Stata Corporation, College Station, TX, USA).

## 3. Results

The percentage trends of BMI categories are reported in Table 1 according to the five wave periods. In particular, between the first and the last wave the percentage of normal weight adolescents showed a 10% decrease in girls of 11- and 12-years-old, while the 13-year-old girls decreased by 3% only. The boys also revealed a greater decrease at 11-years-old and a smaller decrease at 13-years-old. Conversely, the percentage of adolescents with obesity increased approximately three times in 11- and 12-year-old girls and 11-year-old boys, and it doubled in 12- and 13-year-old boys.

The descriptive characteristics of boys and girls are shown in Table 2.

The PF outcome of the subjects are reported in Table 3 and Table 4.

For the 4×5 m test, the best results within all waves were obtained by 13-year-old girls and 12-year-old boys. In particular, girls with obesity had a maximum difference of 0.56 s with respect to normal-weight peers. The normal-weight girls worsened the performance by 0.15, 0.14, and 0.13 s at each wave, respectively, for 11, 12, and 13 year olds, while girls with obesity worsened the performance by 0.22, 0.18, and 0.17 s. Boys showed a mean performance difference of 0.4 s between normal-weight and obese peers with a decrease of 0.1 s in performance per decade for normal-weight adolescents and of 0.15 s for adolescents with obesity.

In the 30 m sprint test, performance remained essentially unchanged along the waves for both male and female, even if some considerations could be reported. Both normal-weight and obese boys aged twelve years old showed a greater decline. In the last period, >2005, 11 and 13-year-old boys with obesity had the greatest performance (5.5% e 3%). In girls with obesity, we found the greatest decline during the >2005 period for 11-year-olds, <1990 period for 12-year-olds, and between the 1991 and the 1995 period for 13-year-olds. In normal-weight girls, performance differences were negligible.

Moreover, the results of the 60 m sprint test were almost stable during the whole period with small performance improvements between the first and the last wave in females of 11-, 12-, and 13-years-old. The most consistent improvement in performance was, however, between the ages of 11 and 12, both for the normal-weight and the obese girls.

Additionally, both normal-weight and obese boys decreased the time spent in the sprint during the whole period by approximately 0.5 s (normal-weight) and 1 s (adolescents with obesity). In girls, the greatest gap between normal-weight adolescents and adolescents with obesity (considering all the periods) was in the 11-year-old age group. 

Specifically, BMI, age, and gender showed significant differences for all of the PF tests (see Table 5).

Table 5 reveals that within BMI categories (normal or with obesity) sex and gender remain crucial factors influencing performance (*p* < 0.001).

A significative decline in the 4×5 m run test (*p* < 0.001) was noted through the years (in particular in the last wave) in both sexes; an increase in the BMI was also noted. While we did not report a decline in the 30 m and the 60 m run tests, in particular we noted a decline in the 4×5 m run test also stratifying for BMI categories in both genders from 1990 to 2010 for normal weight adolescents and from 1995 to 2010 for overweight/obese adolescents.

As reported in Table 5, multivariate analysis showed that the changes in the physical fitness test across years are related to age, sex, and BMI for all the variables (*p* < 0.01).

## 4. Discussion

PF is considered as a marker of health in children and adolescents [7]. Several studies have demonstrated the importance of investigating PF from an early age for a positive relationship between higher levels of PF and the health of children and adolescents and an inverse relationship between PF and the risk of developing CVD [7,16,17]. Prevention and early intervention to promote exercise are mandatory to protect children’s health. We investigated SA trends in adolescents with obesity, showing an undetermined trend across years. In particular, the 4×5 m run test score significantly decreased (*p* < 0.05) in adolescents with overweight/obesity from 1985 to 2010 in both genders, while we did not report a decline in the 30 m and the 60 m run tests. We also noted a decline in the 4×5 m run test score stratifying for BMI categories, in both genders, from 1990 to 2010 for normal weight adolescents and from 1995 to 2010 for adolescents with overweight/obesity. The changes in the PF test results across years are related to age, sex, and BMI, confirming a plausible multifactorial assessment.

The results obtained in the 4×5 m sprint test were in accordance with previous studies that underlined a decreasing performance across years. Ruiz et al. [46] analyzed performances in health-related fitness tests (20-m shuttle run test, standing long jump, and 4×5 m shuttle test) on 214 students aged between 13 and 16 years, they reported the negative and significant relationship between speed and level of PA. As opposed to sedentary children, SA tended to be greater in active children and in males as opposed to females. Koulouvaris et al. studied sprints in boys and girls, aged 5–12 years, reporting significant differences for time in BMI categories [47]; and, Munoz et al. [22], Agha-Alinejad et al. [48], and Trzcińska et al. [49] showed that overweight and obese adolescents had poorer performance in SA tests than their normal-weight peers. Moreover, a previous study by Reisberg et al. (2021) [50] showed a significative relationship between SA and the fat mass index; in their study, at baseline, motor fitness was assessed by the 4×10 m shuttle run test, and a greater SA on the shuttle run test was associated with a lower fat mass index, while improvements in motor fitness were associated with decreases in the fat free mass index. Even though we noted that trends in PF tests are related to different factors, according to the model established by Tomkinson [42,51], we agree that the reported secular trends in PF are caused by a network of social, behavioral, physical, psychosocial, and physiological factors that may also be related to a temporal trend of obesity, including increased sedentary behavior. Since there is no clear evidence for these interactions, especially in the Italian context, we auspicate further investigations aimed to clarify possible causes and solutions. In fact, data on 11-, 13-, and 15-year-old Italian adolescents from the Health Behaviour in School-aged Children (HBSC) World Health Organization (WHO) collaborative cross-national study (2002–2014) [52] indicated a decline in the country-level prevalence of children who achieved at least 60 min of moderate-to-vigorous PA every day or vigorous PA at least four times per week, and an increase in the country-level prevalence of children who used computers recreationally for at least 2 h every weekday. Such trends are supported by Guthold et al. [53] who reported that the country-level prevalence of insufficient PA (i.e., doing less than 60 min of moderate-to-vigorous physical activity every day) increased among Italian adolescents aged 11–17 years between 2001 and 2016.

The results obtained in the 30-m and in the 60-m sprint test were in accordance with a previous Italian study by Lovecchio et al. 2020 [22] that showed a non-significant change in sprint test scores in males and females from 2004 to 2013. This result was also confirmed by another Italian study by Gallotta et al. [54]. Other studies evaluating this test (Greece 1997 to 2007; Spain, 2001 to 2007; Germany 2007–2015) indicated a general improvement in speed performance while others (Lithuania 1992–2002; Portugal 1993–2013) declared a stagnation of results [55]. Additionally, in adolescents with obesity we reported a non-significant change in sprint test scores across years highlighting that excessive body weight does not interfere with short and explosive performance without change of direction as occurs in the 4×5 m sprint test. This unexpected result could be relevant to affirm that adolescents with obesity had worst performance of normal-weight adolescents but remain similar across years. Body weight seems to worsen performance because during direction changes, the augment loads on joints does not permit an explosive response. So, the promotion of exercise focused on specific PF components such as SA could contrast the reduction in the PF performance gap between normal-weight adolescents and those with obesity and, above all, the careful choice of the evaluation battery could be a real action in health education. In fact, as already demonstrated by Lovecchio & Zago [15] in short distance running tests, the difference between normal-weight and obese children is very limited; therefore, the result can be used as an attractive element to promote the performance of adolescents with obesity that experience a more adequate condition. 

Nowadays, there is evidence of a global increase in the BMI of children and adolescents (WHO) and our study also confirmed an increase in adolescents with overweight/obesity from 1984 to 2010 in Italy. Increases in the BMI likely reflect increases in both the fat mass and the fat-free mass. An augmentation in the fat mass could compromise weight-bearing performance, to a greater extent in distance running than in sprint running or explosive jumping, while increases in the fat-free mass should ameliorate performance in tests requiring strength and power, such as sprint running. Tomkinson et al. [56] reported that there is a negative relationship between fat mass and sprint running and there is a stronger positive relationship between fat-free mass and sprint in a large sample of Australian youth. These results suggest that the recent declines in power and speed could have occurred because the positive effects of increasing fat-free mass have not quite matched the negative effect of increasing fat mass. While these data suggest a reason for the recent decline, it is not known whether the secular differences in fat mass and fat-free mass have been consistent over the entire 1984–2010 period. In light of this, a strong and a global analysis could contemporarily use weight and height as affecting the parameter of performance, at least during the pre-adolescent phase [22]. In our results, age and gender are weak influential factors in the PF trend. During the last decades, a secular trend of timing of puberty, particularly in females, was also reported. Puberty [57,58,59] is a combination of physical, physiological, and psychological changes, resulting in physical growth modification, which is a determining factor for assessment of PF; thus, the role of an earlier pubertal timing could be considered. In fact, we can generally see that girls had the worst performance during the whole period near the age of 11, with boys near the age of 13. This result should be further studied considering more precise anamnesis on menarche and the growth stage [60]. Previous studies indicated that higher PF performances were relevant to predicting general well-being in adolescents [6,7] and preventive surveillance of PF performances could help both the clinicians and the trainers to implement strategies to obtain better health outcomes in the young. Moreover, further studies should investigate the influence of sedentary behaviors and the large use of screen devices on PF decline and on health-related outcomes.

While we do not know if our declines in SA performance have evolved beyond 2010, we are certainly encouraged by the release of Italy’s first national guidelines for PA in 2019 [61]. This document supports the National Prevention Plans (2014–2019 and new 2020–2025) [61], which improve health-enhancing PA for children through school policies, planned life skills interventions, active travels to/from school (e.g., the Pedibus), sports participation, and active breaks during lessons. Recent studies have shown that an online training program seems to be an important instrument for maintaining health and fitness in children with overweight/obesity [62,63]. Future studies should evaluate the effectiveness and monitor the progress of implemented PA promotion efforts by examining recent temporal trends in SA levels and in adolescents with overweight/obesity. We must recognize some limitations in our study. No data on pubertal stages were recorded in our subjects; further studies considering also pubertal stages could be useful to clarify the determinant factors of the decrease in performance across years. Similarly, the lack of data on body composition and on lifestyles does not allow a complete interpretation of the data. Finally, all participants were Caucasian and sampled from one school in northern Italy and therefore the results may not be generalizable to other demographic groups or countries where the network of social, behavioral, psychosocial, and psychological factors may substantially differ. Despite these limitations, this is the first study that investigated the SA ability in adolescents with a special focus on young people with obesity, considering a 25-year investigation; and, our results could highlight the necessity for implementing a national surveillance on PF capacities in children and adolescents. 

## 5. Conclusions

In conclusion, fitness tests highlighted differences in normal weight compared to adolescents with overweight/obesity that could be important for health monitoring, at least in a brief sprint performance. Additionally, SA fitness decline is an alarming factor that should generate corrective actions. Our results could support a fruitful body of future work on a larger national scale to better understand the mechanisms of observed trends and ameliorated young health through monitoring PF capacities, especially in adolescents with obesity. 

## Figures and Tables

**Table 1:** Prevalence of normal weight subjects and adolescents with overweight/obesity across years and divided per gender and age.

		Girls						Boys					
		11 y		12 y		13 y		11 y		12 y		13 y	
	Wave	*n*	%	*n*	%	*n*	%	*n*	%	*n*	%	*n*	%
**Normal-weight**	<1990	61	91.04	59	93.65	48	82.76	153	91.07	173	90.10	179	90.86
	1991–1995	95	85.59	121	85.03	128	84.77	112	85.50	134	88.58	109	87.20
	1996–2000	112	86.82	121	87.43	106	83.46	134	84.28	117	82.98	112	78.32
	2001–2005	115	87.12	131	88.92	129	82.69	128	84.77	135	88.24	116	87.88
	>2005	110	73.97	109	77.86	97	82.20	105	73.94	132	80.00	118	80.82
**Overweight/obese**	<1990	6	8.96	4	6.35	10	17.24	15	8.93	19	9.90	18	9.14
	1991–1995	16	14.41	23	15.97	23	15.23	19	14.50	19	12.42	16	12.80
	1996–2000	17	13.18	19	13.57	21	16.54	25	15.72	24	17.02	31	21.68
	2001–2005	17	12.88	18	12.08	27	17.31	23	15.23	18	11.76	16	12.12
	>2005	40	27.03	31	22.14	21	17.80	37	26.06	33	20.00	28	19.18

*n* = number of adolescents; % = percentage on the total.

**Table 2:** Descriptive characteristics of boys and girls.

		BMI ^1^ z-Score		
	Wave	11 y	12 y	13 y
**Girls**	<1990	−0.31 (−0.93–0.36)	−0.40 (−1.00–0.30)	−0.07 (−0.84–0.67)
	1991–1995	−0.16 (−1.15–0.52)	−0.08 (−0.91–0.74)	−0.09 (−0.84–0.67)
	1996–2000	−0.11 (−0.83–0.43)	−0.10 (−0.84–0.57)	−0.16 (−0.82–0.64)
	2001–2005	−0.44 (−1.10–0.60)	−0.38 (−1.11–0.47)	−0.18 (−0.87–0.58)
	>2005	0.26 (−0.47–1.12)	0.04 (−0.55–0.85)	−0.04 (−0.76–0.73)
**Boys**	<1990	−0.36 (−1.12–0.43)	−0.51 (−1.21–0.32)	−0.51 (−1.22–0.33
	1991–1995	−0.15 (−1.07–0.55)	−0.36 (−1.22–0.28)	−0.33 (−0.94–0.38)
	1996–2000	−0.27 (−0.95–0.54)	−0.05 (−0.78–0.73)	−0.18 (−0.86–0.80)
	2001–2005	−0.27 (−0.94–0.52)	−0.36 (−1.11–0.46)	−0.33 (−1.06–0.27)
	>2005	0.05 (−0.85–1.02)	−0.12 (−0.82–0.75)	−0.12 (−0.95–0.62)

All values are shown as median (25th–75th percentiles). ^1^ BMI = Body Mass Index.

**Table 3:** Mean physical fitness score by body weight and wave for girls aged 11–13 y (*n* = 1.739).

	Girls												
		11 y				12 y				13 y			
		Normal-Weight		Obese		Normal-Weight		Obese		Normal-Weight		Obese	
	Wave	Mean	SD	Mean	SD	Mean	SD	Mean	SD	Mean	SD	Mean	SD
**4** **×5-m s**	<1990	7.49	0.37	7.57	0.69	7.29	0.33	7.71	0.90	7.23	0.37	7.50	0.47
	1991–1995	7.56	0.43	7.86	0.44	7.43	0.43	7.48	0.36	7.27	0.42	7.43	0.45
	1996–2000	7.73	0.50	8.29	0.68	7.58	0.56	8.11	0.75	7.27	0.41	7.55	0.36
	2001–2005	7.78	0.55	7.94	0.41	7.51	0.48	8.06	0.62	7.41	0.58	7.74	0.47
	>2005	8.05	0.48	8.46	0.48	7.85	0.51	8.43	0.75	7.75	0.51	8.19	0.51
**30-m s**	<1990	5.48	0.38	5.61	0.58	5.29	0.29	5.87	0.51	5.25	0.32	5.49	0.22
	1991–1995	5.45	0.36	5.77	0.55	5.28	0.45	5.52	0.38	5.14	0.45	5.39	0.45
	1996–2000	5.45	0.37	5.89	0.77	5.28	0.36	5.73	0.50	5.14	0.33	5.52	0.41
	2001–2005	5.45	0.45	5.62	0.32	5.22	0.38	5.61	0.38	5.21	0.42	5.59	0.42
	>2005	5.43	0.37	5.81	0.47	5.25	0.37	5.67	0.44	5.16	0.34	5.50	0.49
**60-m s**	<1990	10.99	1.32	11.35	1.43	10.36	0.71	12.24	1.38	10.24	0.70	10.83	0.53
	1991–1995	10.81	0.85	12.35	2.42	10.31	1.08	10.75	0.78	10.20	1.40	10.60	1.00
	1996–2000	10.77	0.81	11.94	1.67	10.44	1.27	11.35	0.96	9.97	0.80	10.99	1.04
	2001–2005	10.69	1.05	11.04	0.75	10.19	0.90	10.99	0.83	10.14	1.00	10.96	1.06
	>2005	10.48	1.18	11.41	1.09	10.20	0.74	11.29	1.05	10.01	0.80	10.79	1.07

4×5-m = 4×5 m sprint test, s = seconds; 60-m = 60-m sprint test; 30-m = 30-m sprint test.

**Table 4:** Mean physical fitness score by body weight and wave for boys aged 11–13 y (*n* = 2.184).

		Boys											
		11 y				12 y				13 y			
		Normal-Weight		Obese		Normal-Weight		Obese		Normal-Weight		Obese	
	Wave	Mean	SD	Mean	SD	Mean	SD	Mean	SD	Mean	SD	Mean	SD
**4** **×5-m s**	<1990	7.31	0.46	7.63	0.49	7.11	0.54	7.43	0.43	6.87	0.44	7.14	0.27
	1991–1995	7.27	0.35	7.69	0.45	7.09	0.52	7.16	0.61	6.85	0.41	7.22	0.43
	1996–2000	7.42	0.47	7.88	0.57	7.25	0.49	7.54	0.42	6.96	0.44	7.27	0.40
	2001–2005	7.38	0.45	7.95	0.49	7.22	0.47	7.87	0.69	6.93	0.43	7.52	0.41
	>2005	7.76	0.57	8.37	0.71	7.49	0.53	7.96	0.59	7.30	0.54	7.74	0.71
**30-m s**	<1990	5.27	0.33	5.78	0.32	5.04	0.32	5.70	0.40	4.81	0.32	5.24	0.39
	1991–1995	5.29	0.36	5.76	0.36	5.08	0.68	5.49	0.55	4.94	0.54	5.36	0.53
	1996–2000	5.31	0.41	5.67	0.45	5.17	0.40	5.63	0.45	4.89	0.39	5.17	0.53
	2001–2005	5.24	0.35	5.60	0.35	5.05	0.37	5.49	0.84	4.76	0.38	5.18	0.43
	>2005	5.26	0.45	5.91	0.56	5.11	0.47	5.59	0.73	4.84	0.45	5.34	0.71
**60-m s**	<1990	10.29	0.76	11.28	0.63	9.71	0.75	11.11	1.18	9.17	0.74	10.19	0.95
	1991–1995	10.33	0.85	11.48	0.93	10.07	1.56	10.85	1.33	9.49	1.22	10.34	1.13
	1996–2000	10.52	1.65	11.23	1.45	10.11	0.99	11.09	1.01	9.41	0.92	10.20	1.28
	2001–2005	10.22	0.86	11.04	0.76	9.76	0.81	10.92	1.95	9.10	0.76	10.11	0.91
	>2005	10.20	0.97	11.56	1.20	9.92	0.98	10.84	1.62	9.32	0.80	10.30	1.58

4×5-m= 4×5 m sprint test, s = seconds; 60-m = 60-m sprint test; 30-m = 30-m sprint test.

**Table 5:** Multivariate analysis of the whole sample.

		4×5-m (s)	60-m (s)	30-m (s)
**All the sample**	**BMI ^3^**	<0.001	<0.001	<0.001
		0.29 (0.25–0.34)	0.68 (0.59–0.78)	0.06 (0.09–0.19)
	**1991–1995**	0.031	0.105	0.924
		0.06 (0.01–0.34)	0.09 (−0.02–0.19)	0.00 (0.40–0.44)
	**1996–2000**	<0.001	0.772	0.444
		0.16 (0.10–0.21)	0.02 (−0.10–0.14)	−0.19 (−0.07–0.03)
	**2001–2005**	<0.001	0.469	0.843
		0.18 (0.12–0.24)	−0.04 (−0.16–0.75)	−0.00 (−0.05–0.04)
	**>2005**	<0.001	0.691	0.953
		0.49 (0.43–0.55)	−0.02 (−0.15–0.10)	0.00 (−0.04–0.05)
	**Age**	<0.001	<0.001	<0.001
		−0.20 (−0.21–0.19)	−0.46 (−0.21–0.41)	−0.18 (−0.18–0.16)
	**Gender**	<0.001	<0.001	0.02
		−0.29 (–0.33–0.25)	−0.45 (–0.53–0.37)	−0.07 (–0.14–0.008)
**Normal** **weight**	**Age**	<0.001	<0.001	<0.001
		−0.20 (−0.21–0.18)	−0.43 (−0.46–0.40)	−0.18 (−0.19–0.17)
	**Gender**	<0.001	<0.001	<0.001
		−0.31 (–0.35–0.26)	−0.49 (–0.58–0.40)	−0.21 (–0.24–0.17)
	**1991–1995**	0.046	0.149	0.993
		0.06 (0.00–0.11)	0.08 (−0.03–0.20)	0.00 (0.04–0.05)
	**1996–2000**	<0.001	0.462	0.565
		0.15 (0.09–0.21)	0.05 (−0.08–0.17)	−0.01 (−0.07–0.04)
	**2001–2005**	<0.001	0.552	0.751
		0.15 (0.09–0.21)	−0.04 (−0.17–0.09)	−0.01 (−0.06–0.04)
	**>2005**	<0.001	0.292	0.247
		0.44 (0.37–0.50)	−0.07 (−0.20–0.06)	0.03 (−0.09–0.02)
**Obese**	**Age**	<0.001	<0.001	<0.001
		−0.24 (−0.28–0.20)	−0.48 (−0.57–0.39)	−0.21 (−0.24–0.18)
	**Gender**	<0.001	<0.001	0.035
		−0.24 (–0.35–0.13)	−0.34 (–0.57–0.10)	−0.11 (–0.21–0.01)
	**1991–1995**	0.795	0.696	0.586
		−0.02 (−0.20–0.15)	0.08 (−0.31–0.46)	0.04 (−0.11–0.19)
	**1996–2000**	<0.011	0.654	0.775
		0.23 (0.05–0.41)	0.09 (−0.30–0.48)	−0.02 (−0.13–0.18)
	**2001–2005**	0.001	0.700	0.868
		0.30 (0.12–0.81)	−0.08 (−0.47–0.32)	−0.13 (−0.14–0.17)
	**>2005**	<0.001	0.773	0.301
		0.63 (0.46–0.81)	−0.06 (−0.32–0.44)	0.08 (−0.07–0.24)

^3^ BMI = Body Mass Index; 4×5-m = 4×5 m sprint test, s = seconds; 60-m = 60-m sprint test; 30-m = 30-m sprint test.

## Data Availability

All data can be obtained from the corresponding author upon reasonable request.

## References

[1] World Health Organization Noncommunicable Diseases: Childhood Overweight and Obesity. https://www.who.int/dietphysicalactivity/childhood/en/.

[2] Istituto Superiore di Sanità OKkio Alla SALUTE 2022. https://www.epicentro.iss.it/okkioallasalute/.

[3] Morales Camacho W.J., Molina Díaz J.M., Plata Ortiz S., Plata Ortiz J.E., Morales Camacho M.A., Calderón B.P. (2019). Childhood Obesity: Aetiology, Comorbidities, and Treatment. Diabetes Metab. Res. Rev..

[4] Simmonds M., Llewellyn A., Owen C.G., Woolacott N. (2016). Predicting Adult Obesity from Childhood Obesity: A Systematic Review and Meta-Analysis. Obes. Rev..

[5] Marcus B.H., Williams D.M., Dubbert P.M., Sallis J.F., King A.C., Yancey A.K., Franklin B.A., Buchner D., Daniels S.R., Claytor R.P. (2006). Physical Activity Intervention Studies: What We Know and What We Need to Know: A Scientific Statement from the American Heart Associ.iation Council on Nutrition, Physical Activity, and Metabolism (Subcommittee on Physical Activity); Council on Cardiovascular Disease in the Young; and the Interdisciplinary Working Group on Quality of Care and Outcomes Research. Circulation.

[6] Elagizi A., Kachur S., Carbone S., Lavie C.J., Blair S.N. (2020). A Review of Obesity, Physical Activity, and Cardiovascular Disease. Curr. Obes. Rep..

[7] Ortega F.B., Ruiz J.R., Castillo M.J., Sjöström M. (2008). Physical Fitness in Childhood and Adolescence: A Powerful Marker of Health. Int. J. Obes..

[8] Pescatello L.S., Hennessy E.A., Katzmarzyk P.T., Kraus W.E., Fish A.F., Craft L.L., Johnson B.T. (2021). Best Practices for Meta-Reviews in Physical Activity and Health Research: Insights From the Physical Activity Guidelines for Americans Advisory Committee Scientific Report. J. Phys. Act. Health.

[9] Powell K.E., King A.C., Buchner D.M., Campbell W.W., DiPietro L., Erickson K.I., Hillman C.H., Jakicic J.M., Janz K.F., Katzmarzyk P.T. (2018). The Scientific Foundation for the Physical Activity Guidelines for Americans, 2nd Edition. J. Phys. Act. Health.

[10] Valerio G., Maffeis C., Saggese G., Ambruzzi M.A., Balsamo A., Bellone S., Bergamini M., Bernasconi S., Bona G., Calcaterra V. (2018). Diagnosis, Treatment and Prevention of Pediatric Obesity: Consensus Position Statement of the Italian Society for Pediatric Endocrinology and Diabetology and the Italian Society of Pediatrics. Ital. J. Pediatr..

[11] Vandoni M., Lovecchio N., Carnevale Pellino V., Codella R., Fabiano V., Rossi V., Zuccotti G.V., Calcaterra V. (2021). Self-Reported Physical Fitness in Children and Adolescents with Obesity: A Cross-Sectional Analysis on the Level of Alignment with Multiple Adiposity Indexes. Children.

[12] Vandoni M., Calcaterra V., Pellino V.C., De Silvestri A., Marin L., Zuccotti G.V., Tranfaglia V., Giuriato M., Codella R., Lovecchio N. (2021). “Fitness and Fatness” in Children and Adolescents: An Italian Cross-Sectional Study. Children.

[13] Ortega F.B., Ruiz J.R., España-Romero V., Vicente-Rodriguez G., Martínez-Gómez D., Manios Y., Béghin L., Molnar D., Widhalm K., Moreno L.A. (2011). The International Fitness Scale (IFIS): Usefulness of Self-Reported Fitness in Youth. Int. J. Epidemiol..

[14] Giuriato M., Lovecchio N., Fugiel J., Lopez Sanchez G.F., Pihu M., Emeljanovas A. (2020). Enjoyment and Self-Reported Physical Competence According to Body Mass Index: International Study in European Primary School Children. J. Sports Med. Phys. Fit..

[15] Lovecchio N., Zago M. (2019). Fitness Differences According to BMI Categories: A New Point of View. J. Sports Med. Phys. Fit..

[16] Imboden M.T., Harber M.P., Whaley M.H., Finch W.H., Bishop D.L., Kaminsky L.A. (2018). Cardiorespiratory Fitness and Mortality in Healthy Men and Women. J. Am. Coll. Cardiol..

[17] García-Hermoso A., Ramírez-Campillo R., Izquierdo M. (2019). Is Muscular Fitness Associated with Future Health Benefits in Children and Adolescents? A Systematic Review and Meta-Analysis of Longitudinal Studies. Sports Med..

[18] Högström G., Nordström A., Nordström P. (2016). Aerobic Fitness in Late Adolescence and the Risk of Early Death: A Prospective Cohort Study of 1.3 Million Swedish Men. Int. J. Epidemiol..

[19] Johansson L., Brissman M., Morinder G., Westerståhl M., Marcus C. (2020). Reference Values and Secular Trends for Cardiorespiratory Fitness in Children and Adolescents with Obesity. Acta. Paediatr..

[20] Smith J.J., Eather N., Morgan P.J., Plotnikoff R.C., Faigenbaum A.D., Lubans D.R. (2014). The Health Benefits of Muscular Fitness for Children and Adolescents: A Systematic Review and Meta-Analysis. Sports Med..

[21] Đurić S., Sember V., Starc G., Sorić M., Kovač M., Jurak G. (2021). Secular Trends in Muscular Fitness from 1983 to 2014 among Slovenian Children and Adolescents. Scand. J. Med. Sci. Sports.

[22] Giuriato M., Codella R., Lovecchio N., Carnevale Pellino V., Vandoni M., Nevill A.M. (2021). Speed Agility Trends in Children According to Growth. Ann. Hum. Biol..

[23] Carnevale Pellino V., Giuriato M., Ceccarelli G., Codella R., Vandoni M., Lovecchio N., Nevill A.M. (2020). Explosive Strength Modeling in Children: Trends According to Growth and Prediction Equation. Appl. Sci..

[24] Howley E.T. (2001). Type of Activity: Resistance, Aerobic and Leisure versus Occupational Physical Activity. Med. Sci. Sports Exerc..

[25] Sheppard J.M., Young W.B. (2006). Agility Literature Review: Classifications, Training and Testing. J. Sports Sci..

[26] Ortega F.B., Cadenas-Sánchez C., Sánchez-Delgado G., Mora-González J., Martínez-Téllez B., Artero E.G., Castro-Piñero J., Labayen I., Chillón P., Löf M. (2015). Systematic Review and Proposal of a Field-Based Physical Fitness-Test Battery in Preschool Children: The PREFIT Battery. Sports Med..

[27] Lovecchio N., Giuriato M., Carnevale Pellino V., Valarani F., Codella R., Vandoni M. (2020). Italian Physical Fitness Decline: A True Fact or a Mindset? A 10-Year Observational Perspective Study. Int. J Environ. Res. Public Health.

[28] Ogden C.L., Carroll M.D., Kit B.K., Flegal K.M. (2014). Prevalence of Childhood and Adult Obesity in the United States, 2011–2012. JAMA.

[29] Fan H., Zhang X. (2020). Alarming Trends in Severe Obesity in Chinese Children from 1991 to 2015. Child. Obes..

[30] Eberhardt T., Niessner C., Oriwol D., Buchal L., Worth A., Bös K. (2020). Secular Trends in Physical Fitness of Children and Adolescents: A Review of Large-Scale Epidemiological Studies Published after 2006. Int. J Environ. Res. Public Health.

[31] Fühner T., Kliegl R., Arntz F., Kriemler S., Granacher U. (2021). An Update on Secular Trends in Physical Fitness of Children and Adolescents from 1972 to 2015: A Systematic Review. Sports Med..

[32] Tomkinson G., Hamlin M., Olds T. (2006). Secular Changes in Anaerobic Test Performance in Australasian Children and Adolescents. Pediatric Exerc. Sci..

[33] Baquet G., van Praagh E., Berthoin S. (2003). Endurance Training and Aerobic Fitness in Young People. Sports Med..

[34] Ward D., Evans R. (1995). Physical Activity, Aerobic Fitness and Obesity in Children. Med. Exerc. Nutr. Health.

[35] Bandini L.G., Schoeller D.A., Dietz W.H. (1990). Energy Expenditure in Obese and Nonobese Adolescents. Pediatr. Res..

[36] Mei Z., Grummer-Strawn L.M. (2007). Standard Deviation of Anthropometric Z-Scores as a Data Quality Assessment Tool Using the 2006 WHO Growth Standards: A Cross Country Analysis. Bull. World Health Organ..

[37] (2013). JAVA Declaration of Helsinki World Medical Association Declaration of Helsinki. Bull. World Health Organ..

[38] da Silva V., Vieira F. (2020). International Society for the Advancement of Kinanthropometry (ISAK) Global: International Accreditation Scheme of the Competent Anthropometrist. Rev. Bras. De Cineantropom. Desempenho Hum..

[39] Skowronski W., Horvat M., Nocera J., Roswal G., Croce R. (2009). Eurofit Special: European Fitness Battery Score Variation among Individuals with Intellectual Disabilities. Adapt. Phys. Activ. Q..

[40] Fjørtoft I., Pedersen A.V., Sigmundsson H., Vereijken B. (2011). Measuring Physical Fitness in Children Who Are 5 to 12 Years Old with a Test Battery That Is Functional and Easy to Administer. Phys. Ther..

[41] Miežienė B., Česnaitienė V.J., Emeljanovas A., Fjortoft I., Kjønniksen L., Kreivytė R., Tilindienė I., Zaičenkovienė K. (2017). Functional Physical Fitness in 7–10-Year-Old School Children in Lithuania. Pilot Study. Balt. J. Sport Health Sci..

[42] Tomkinson G., Olds T. (2008). Field Tests of Fitness. Paediatr. Exerc. Sci. Med..

[43] Vicente-Rodríguez G., Rey-López J.P., Ruíz J.R., Jiménez-Pavón D., Bergman P., Ciarapica D., Heredia J.M., Molnar D., Gutierrez A., Moreno L.A. (2011). Interrater Reliability and Time Measurement Validity of Speed-Agility Field Tests in Adolescents. J. Strength Cond. Res..

[44] Hanssen-Doose A., Niessner C., Oriwol D., Bös K., Woll A., Worth A. (2021). Population-Based Trends in Physical Fitness of Children and Adolescents in Germany, 2003-2017. Eur. J. Sport Sci..

[45] Dyrstad S.M., Berg T., Tjelta L.I. (2012). Secular Trends in Aerobic Fitness Performance in a Cohort of Norwegian Adolescents. Scand J. Med. Sci. Sports.

[46] Ruiz J.R., Castro-Piñero J., España-Romero V., Artero E.G., Ortega F.B., Cuenca M.M., Jimenez-Pavón D., Chillón P., Girela-Rejón M.J., Mora J. (2011). Field-Based Fitness Assessment in Young People: The ALPHA Health-Related Fitness Test Battery for Children and Adolescents. Br. J. Sports Med..

[47] Koulouvaris P., Tsolakis C., Tsekouras Y.E., Donti O., Papagelopoulos P.J. (2018). Obesity and Physical Fitness Indices of Children Aged 5-12 Years Living on Remote and Isolated Islands. Rural Remote Health.

[48] Agha-Alinejad H., Farzad B., Salari M., Kamjoo S., Harbaugh B.L., Peeri M. (2015). Prevalence of Overweight and Obesity among Iranian Preschoolers: Interrelation nship with Physical Fitness. J. Res. Med. Sci..

[49] Trzcińska D., Olszewska E., Tabor P. (2009). Physical fitness and body posture of 7-years-old children with extreme somatic parameters. Pediatr. Endocrinol. Diabetes Metab..

[50] Reisberg K., Riso E.-M., Jürimäe J. (2021). Physical Fitness in Preschool Children in Relation to Later Body Composition at First Grade in School. PLoS ONE.

[51] Tomkinson G.R., Olds T.S. (2007). Secular Changes in Pediatric Aerobic Fitness Test Performance: The Global Picture. Med. Sport Sci..

[52] Epidemiology for Public Health Health Behaviour in School-Aged Children (HBSC). https://www.epicentro.iss.it/en/hbsc/.

[53] Guthold R., Stevens G.A., Riley L.M., Bull F.C. (2018). Worldwide Trends in Insufficient Physical Activity from 2001 to 2016: A Pooled Analysis of 358 Population-Based Surveys with 1·9 Million Participants. Lancet Glob. Health.

[54] Gallotta M.C., Marchetti R., Baldari C., Guidetti L., Pesce C. (2009). Linking Coordinative and Fitness Training in Physical Education Settings. Scand. J. Med. Sci. Sports.

[55] Masanovic B., Gardasevic J., Marques A., Peralta M., Demetriou Y., Sturm D.J., Popovic S. (2020). Trends in Physical Fitness Among School-Aged Children and Adolescents: A Systematic Review. Front. Pediatr..

[56] Tomkinson G.R., Olds T.S. (2007). Secular Changes in Aerobic Fitness Test Performance of Australasian Children and Adolescents. Med. Sport Sci..

[57] Delemarre-van de Waal H. (2005). Secular Trend of Timing of Puberty. Endocr. Dev..

[58] Karlberg J. (2002). Secular Trends in Pubertal Development. Horm Res..

[59] Eckert-Lind C., Busch A.S., Petersen J.H., Biro F.M., Butler G., Bräuner E.V., Juul A. (2020). Worldwide Secular Trends in Age at Pubertal Onset Assessed by Breast Development Among Girls: A Systematic Review and Meta-Analysis. JAMA Pediatr..

[60] Malina R.M., Rogol A.D., Cumming S.P., Coelho e Silva M.J., Figueiredo A.J. (2015). Biological Maturation of Youth Athletes: Assessment and Implications. Br. J. Sports Med..

[61] Ministry of Health Italian National Prevention Plan. https://www.google.com/url?sa=t&rct=j&q=&esrc=s&source=web&cd=&ved=2ahUKEwjTzuunqaf1AhVGRPEDHXP7D1QQFnoECAMQAQ&url=https%3A%2F%2Fwww.ccm-network.it%2Fpagina.jsp%3Fid%3Dnode%2F316%26lingua%3Denglish&usg=AOvVaw3Ejk-WRlI9q4RDUf5bf55l.

[62] Vandoni M., Codella R., Pippi R., Carnevale Pellino V., Lovecchio N., Marin L., Silvestri D., Gatti A., Magenes V.C., Regalbuto C. (2021). Combatting Sedentary Behaviors by Delivering Remote Physical Exercise in Children and Adolescents with Obesity in the COVID-19 Era: A Narrative Review. Nutrents.

[63] Calcaterra V., Verduci E., Vandoni M., Rossi V., Di Profio E., Carnevale Pellino V., Tranfaglia V., Pascuzzi M.C., Borsani B., Bosetti A. (2021). Telehealth: A Useful Tool for the Management of Nutrition and Exercise Programs in Pediatric Obesity in the COVID-19 Era. Nutrents.

